# Domain-Specific Expectations in Music Segmentation

**DOI:** 10.3390/brainsci9070169

**Published:** 2019-07-17

**Authors:** Susana Silva, Carolina Dias, São Luís Castro

**Affiliations:** Center for Psychology at University of Porto (CPUP), Faculty of Psychology and Education Sciences, 4200-135 Porto, Portugal

**Keywords:** Prosody, Phrasing, Perception, Melody

## Abstract

The acoustic cues that guide the assignment of phrase boundaries in music (pauses and pitch movements) overlap with those that are known for speech prosody. Based on this, researchers have focused on highlighting the similarities and neural resources shared between music and speech prosody segmentation. The possibility that music-specific expectations add to acoustic cues in driving the segmentation of music into phrases could weaken this bottom-up view, but it remains underexplored. We tested for domain-specific expectations in music segmentation by comparing the segmentation of the same set of ambiguous stimuli under two different instructions: stimuli were either presented as speech prosody or as music. We measured how segmentation differed, in each instruction group, from a common reference (natural speech); thus, focusing on how instruction affected delexicalization effects (natural speech vs. transformed versions with no phonetic content) on segmentation. We saw interactions between delexicalization and instruction on most segmentation indices, suggesting that there is a music mode, different from a speech prosody mode in segmentation. Our findings highlight the importance of top-down influences in segmentation, and they contribute to rethinking the analogy between music and speech prosody.

## 1. Introduction

Most speech listeners and music listeners segment the auditory input into phrase-like units [1,2,3,4]. In both domains, listeners detect phrase boundaries as the input unfolds. This leads to the possibility of building the segmentation map of an utterance or a music piece, defining how many phrases were heard, whether they were short, long, regular or irregular in length, and how they relate to each other. Language and music users have ways of emphasizing their intended segmentation maps (phrase boundary locations) using specific graphic signs in printed versions of language and music. In written language, the intended segmentation map of an utterance is sometimes achieved by printed punctuation marks [5,6]. Printed music does not have a mandatory analogue of punctuation marks to signal the presence of intended phrase boundaries. Slurs are perhaps the most obvious sign of intended segmentation maps, although other markers such as pause signs can also be used [7,8].

In speech research, the idea of segmentation map, a set of individual choices regarding segmentation, has been implemented mostly with pairs of syntactically ambiguous sentences [8,9,10], holding more than one meaning depending on how they are parsed. The way that participants judge and understand such speech materials is then taken as an index of their segmentation choices. While traditional behavioral approaches only allowed delayed (post-exposure) judgements, more recent techniques such as eye-tracking [11,12] or EEG [8,10] popularized the online monitoring of speech segmentation, often affording the tracking of participants’ revisions of their initial segmentation choices [13]. Online monitoring techniques have also increased the interest in music segmentation (e.g., [14]). In the present study, we used a simple behavioral online monitoring approach to both speech and music segmentation maps, which consisted of asking participants to press a key every time they heard a phrase ending.

The segmentation of speech into phrase-like units depends not only on linguistic content (lexico–syntactic structure), but also on the paralinguistic intonation patterns of speech prosody (e.g., [1]), which define intonational phrases. The perception of intonation patterns per se, regardless of interactions with linguistic content, is driven by low-level acoustic cues such as changes in pitch, duration, or the presence of silence. In this sense, it is possible to view the segmentation of intonation-related speech prosody (speech prosody hereafter) as a bottom-up process, i.e., as a process where extra-perceptual factors like previous expectations of what an intonational phrase should be do not play a major role (see [15] for a discussion on top-down vs. bottom-up). Pitch deflections and pauses are acoustic cues that play an important role in driving both the segmentation of music [3,14] and that of speech prosody [16,17,18]. Does it follow that music is segmented the same way as speech prosody? The answer depends on how we assume that music segmentation is driven: if it is driven by acoustic cues, as in speech prosody, the answer is yes. If it is driven both by acoustic cues plus music-specific expectations (i.e., an idea of what a musical phrase is), the answer is no. The literature is mixed on this matter, as we will see below.

The idea that boundary assignment in music is driven solely by acoustic cues, which we will refer to as a bottom-up view on auditory segmentation, is present in the literature on the Closure Positive Shift (CPS) event-related potential. The CPS is an electrophysiological marker of phrase boundary perception, which has been found for speech [8,10,19], delexicalized (hummed) speech [20] and music [14,21,22,23] with little morphological variation across the three [20,24]. The bottom line of the CPS approach is that segmentation shares neural resources across music and speech prosody, and a strong motivation for these studies has been the fact that the same type of segmentation cues (pitch deflections, pauses) can be detected in both domains [14]. CPS studies have focused on the acoustic features that characterize musical and prosodic segmentation points (music phrases vs. intonational phrases). These are expected to elicit a brain response corresponding to boundary detection, with little effects of prior knowledge or contextual aspects. An implication of this view is that the segmentation map of a music piece can be similar to the segmentation map of a sample of speech prosody, provided that both have the same acoustic boundary cues at the same time points.

The alternative view, which we will refer to as the *top-down view*, emphasizes the role of expectations in suppressing or counteracting acoustic boundary cues. For instance, it has been admitted that music-specific expectations can make the listener search for four-bar structures when judging whether a musical phrase has ended or not [22,23], possibly overriding pauses within the four-bar phrase. The top-down view also relates to the idea that music segmentation may rely on more global cues than the segmentation of speech prosody [25]; such cues extending in time beyond the limits of a local boundary mark such as a pause and requiring integration. In contrast to the bottom-up view, one should expect here that equivalent boundary cues in music and speech prosody would not lead to equivalent segmentation maps, since segmentation options would depend on additional music-specific top-down influences. To our knowledge, neither this top-down-based hypothesis nor its bottom-up alternative have been subject to testing.

In the present paper, we tested whether music segmentation into phrases is driven by music-specific expectations that add to the acoustic cues used to segment speech prosody into intonational phrases. Thus, we tested for a top-down view on music segmentation. To that end, we compared participants’ segmentation maps of a single set of ambiguous auditory stimuli, which were either presented as music or as speech prosody. We manipulated only the instruction, inducing different processing modes on the very same acoustic materials: top-down expectations and bottom-up processing for music, against bottom-up processing (only) for speech prosody vs. no additional expectations for speech prosody.

The ambiguous stimuli were obtained by an audio-to-MIDI conversion of natural speech, resulting in pitch-and-rhythm auditory streams deprived of linguistic content. For convenience of expression, we will refer to these wordless auditory streams as delexicalized versions, even though the difference between them and natural speech lies, strictly speaking, at the phonetic level rather than just the lexical one. Due to the algorithms involved in the audio-to-MIDI conversion of natural speech (see methods), two types of data (speech prosody) distortions were expected: first, continuous pitch would be converted into discontinuous pitch (octave divided into 12-semitone intervals), lending a music-like character to these ambivalent streams; second, timing-related information concerning speech syllables might not be integrally preserved, although an approximation was expected. The first type of speech prosody distortion (discontinuous pitch) was necessary to keep the credibility of the music instruction. The second type of distortion created additional differences between delexicalized versions and the original speech signal, such that the former were, strictly speaking, delexicalized and modified. Nevertheless, delexicalized versions contained the pitch-and-timing information listeners use for processing speech prosody, with pitch and timing value-ranges reflecting the ones that occur in natural language. In this sense, we considered our delexicalized versions to be representative of speech prosody, even though they were not an exact copy of the speech prosody patterns that generated them.

In order to minimize participants’ awareness of our experimental manipulation, instruction was set as a between-subjects factor. To circumvent the risk of imperfect group matching inherent to a between-subjects approach, we sought for a common reference (baseline) in the two groups, against which we analyzed participants’ segmentation maps of ambiguous, delexicalized stimuli. The common reference we used was natural speech. Therefore, we collected the segmentation maps of a single set of delexicalized (ambiguous) stimuli (i.e., speech without lexical content) of two groups of participants receiving different types of instruction (speech prosody – “This is prosody” vs. music – “This is music”), as well as the segmentation maps of their natural-speech counterparts, in which case the instruction for segmentation was common to both groups (“This is speech”). We then focused on determining whether delexicalization effects (natural speech vs. delexicalized versions, within-subjects factor) were equivalent under music vs. speech prosody instructions, thus probing between-subjects instruction effects with the benefit of a baseline. Similar deviations from the natural-speech baseline (similar delexicalization effects) across instruction conditions (delexicalized presented as music vs. delexicalized presented as speech prosody) would indicate that music participants adopted segmentation approaches to delexicalized versions similar to those of speech prosody participants. In this case, there would be no reason to admit that there are music-specific expectations in music segmentation. By contrast, different deviations from baseline (different delexicalization effects) would indicate that music participants adopted segmentation approaches to delexicalized versions differing from those of speech prosody participants. In this case, music-specific expectations could be considered real.

The existence of delexicalization effects was a precondition to the goal of comparing such effects across instruction conditions. Delexicalization effects were expected under the speech prosody instruction, at least for one reason: it is known that lexicality – the presence vs. absence of lexical content - affects speech segmentation, in the sense that lexical information may override prosodic boundary markers in phrase boundary assignment [26,27,28] and the so-called *linguistic bias* ([29], see also [30] for a similar phenomenon in word segmentation) emerges (cf. [31]). For instance, Buxó-Lugo and Watson [26] found that listeners consistently report hearing more boundaries at syntactically licensed locations than at syntactically unlicensed locations, even when the acoustic evidence for an intonational boundary was controlled. Cole, Mo and Baek [28] analyzed the predictors of phrase boundary assignment, and found syntactic structure to be the strongest one, winning over prosodic cues. Meyer and colleagues [29] found that 2-phrase prosodic sentences with 2-phrase lexical groups lead to segmentation in 2 phrases, but 1-phrase prosodic sentences do not necessarily lead to a single phrase when there are two lexical groups. In the latter case, an electrophysiological marker of the linguistic bias is visible. On the other hand, the existence of delexicalization effects was a precondition, but not a target of this study, and this is why we did not discuss delexicalization effects per se. Instead, our question was whether the delexicalization effect tested under the music instruction would, or would not, parallel the delexicalization effect under the speech prosody instruction - in other words, if delexicalization would interact with instruction in the generation of segmentation maps.

In our approach, we characterized segmentation maps from two different viewpoints: segment length (correlated with the number of segments), and the matching with predefined segmentation models. Interactions between delexicalization and instruction on any of these measures would indicate music-specific expectations.

## 2. Materials and Methods

### 2.1. Participants

Seventy participants took part in the experiment. Half (*n* = 35) were assigned to the speech instruction (31 women), and the other half to the music instruction (27 women). There was no evidence of significant differences between the two groups concerning age (*M* ± *SD*: 20.54 ± 2.85 for speech, 20.28 ± 1.52 for music; *t*(68) = 0.47, *p* >0.64, *d* = 0.12) and musical training (11 participants in the speech condition had 3.27 ± 2.24 years of training, ten in the music condition with 3.90 ± 2.46; *t*(68) = −0.17, *p* >0.86, *d* = −0.04). All participants had normal hearing. None reported psychiatric or neurological disorders. Participants signed informed consent, according to the Declaration of Helsinki.

### 2.2. Stimuli

Stimulus materials consisted of natural speech samples and delexicalized versions of these (see Supplementary materials). The latter were presented under two different instructions (speech prosody vs. music) but they were physically the same. We used five different samples of natural speech. In order to maximize prosodic spontaneity, we selected these samples from available personal and media recordings instead of laboratory recordings. Each sample contained an utterance, combining full sentences that were semantically related (see Appendix A for transcriptions and sentence structure). Four utterances were spoken by men, and one by a woman. Stimulus 1 contained the online description of a short movie that was being watched by the speaker; stimulus 2 was a fragment of an interview; stimulus 3 and 4 were poems recorded by a famous Portuguese diseur; stimulus 5 was an excerpt from a news broadcast. Stimuli were similar in length (~60 sec., see Table 1), and they were all normalized to 70 dB rms.

To create delexicalized versions, natural speech samples were converted to MIDI with software Live 9 (www.ableton.com), using a bass timbre and settings for monophonic stimuli. This audio-to-MIDI conversion software detects stable-pitch fragments preceded by transients (an attack), disregarding intensity information. When dealing with music audio, the software searches for music notes. In speech-related audio, it should detect syllable-like events.

As shown in Table 1, pitch mean and standard deviation were preserved after audio-to-MIDI conversion (Wilcoxon signed rank tests: *Z* = 0, *p* = 0.059 for mean pitch; *Z* = 2, *p* >0.56 for standard deviation of pitch). In delexicalized (discrete pitch) versions, the pitch change rate was close to the syllable rate of speech (3–4 syllables per second, see [32,33]), supporting the idea that the algorithm captured syllable-like units As for the proportion of silences, it was apparently higher in delexicalized versions, but statistical tests did not confirm this (*Z* = 13, *p* >0.13).

### 2.3. Procedure

We started the experiment with auditory reaction time measurements. Participants heard a series of beeps, among which there was a human voice pronouncing a syllable. They were asked to press a key as soon as they heard the human voice. The purpose of these measurements was to provide a participant-specific correction for reaction times (time between perception and key press) for the task of detecting phrase endings that would be requested in the experiment.

All participants were first exposed to the five delexicalized stimuli. Those under the speech instruction were told that the stimuli were derived from real speech, thus containing “the melody of speech, without the words”. Participants under the music instruction were told that stimuli were “excerpts of contemporary music”. All participants were asked to press the space bar of the computer keyboard every time they perceived a phrase ending. Before the experimental trials, all were given a brief explanation of the concept of phrase (“a speech/music fragment, with a beginning and an end”), followed by a demonstration of a possible way of segmenting either speech prosody (speech instruction) or music (music instruction) into phrases. In these examples, we defined segments with similar length across instructions (6 sec. for speech prosody, 7 sec. for music). Given that the concept of music phrase is not trivial among non-experts, we told music-instruction participants that music phrases “were the equivalent of speech phrases, in that they defined unitary fragments”. We stressed that there were no wrong answers. Participants were given one practice trial, either with a delexicalized utterance (speech instruction) or with a music excerpt (music instruction) and then they proceeded into the experimental trials. Each trial consisted of one stimulus to be segmented. Since segmentation was made online, they were unable to go back for corrections. Therefore, we gave participants a second chance: each stimulus was presented twice in succession, and participants did the segmentation on both (5 x 2 trials). Only the second presentation of each stimulus was considered in the analyses. We presented stimuli no more than twice in order to keep the experiment short enough to avoid fatigue.

After segmenting the five delexicalized stimuli, participants were asked to do the same on the 5 × 2 natural speech counterparts. They were informed that they would listen to “normal speech” and they should, again, press the space bar whenever they sensed the phrase had just ended. Participants were not informed that delexicalized and natural speech had the same source. We created three different versions of the experiment, in order to counterbalance the order of presentation of the five stimuli (1-2-3-4-5; 1-4-5-2-3; 4-1-5-3-2). In each version, stimulus order was common to delexicalized and lexicalized sets. Thus, in version 1, participants heard 1-2-3-4-5 delexicalized and then 1-2-3-4-5 lexicalized. We did so in order to keep delexicalized and lexicalized conditions as equivalent as possible.

At the end of the experiment, participants were given a questionnaire where they rated the level of confidence in their segmentation responses for each block (delexicalized vs natural speech) on a 5-point scale and made any comments they wished to. Stimulus delivery was made with Presentation software (www.neurobs.com, v. 20). The experiment lasted about 40 minutes.

### 2.4. Segmentation Models

Prior to the analysis, we defined virtual segmentation points in each stimulus according to four theoretical models, each model based on a different segmentation cue: Pause, Pitch break, Pitch rise and Pitch drop. The adopted models intended to explore the idea of pauses and pitch movements such as low-level acoustic cues subtending both speech prosody and music segmentation (see Introduction section). Considering the possibility that music segmentation may rely more on global cues (see Introduction section) than local boundary marks, two models targeted local cues (Pauses and Pitch breaks), and two targeted global cues (Pitch rises and Pitch drops).

Pauses and Pitch breaks were considered as local cues, in the sense that they included a restricted number of events (silence onset/offset, sudden pitch change), which unfolded within a short time-window. Based on a preliminary inspection of our five natural speech stimuli, we defined Pauses as silent periods longer than 200 ms. The onset of the Pause was considered the segmentation point. Pitch breaks were marked if two consecutive pitch values that were separated by a silence (shorter than 200 ms, the threshold for pause) differed by more than one standard deviation of the stimulus mean pitch. The onset of the second pitch value was set as the segmentation point. Note that the perception of pitch breaks is necessarily context-dependent, since pitch is continuously changing, and we are focusing on salient pitch breaks, which depend on the overall pitch context. However, the break per se (two different pitch values, separated by a short pause) occurs in a short time window. This is the reason why we considered pitch break as a local cue.

Pitch rises and Pitch drops were viewed as global cues, since they require the integration of multiple (pitch) values across time, and they tend to occur within larger time windows. Pitch rises and Pitch drops were defined as unidirectional pitch movements. Since Pitch drops are more common in natural speech, given the F0 decline phenomenon ([34,35], a universal tendency for pitch to drop across sentences, we used more restrictive criteria for Pitch drops than for Pitch rises. Pitch rises and drops should be either wide in pitch range (at least one third of global pitch range) or long-lasting (minimum 500 ms for pitch rise, and 1000 ms for pitch drops). For Pitch drops, we set the additional criterion that pitch should reach a low-frequency range, namely half a standard deviation from the global mean pitch. Small pitch deflections up to 250/200 ms were allowed within Pitch rise/drop segments, as well as pauses up to 200 ms. The offset of pitch movements (rises or drops) corresponded to the segmentation point. Pitch drops or rises not complying with these criteria were not used as virtual segmentation points of any kind.

When Pauses coexisted with Pitch breaks, rises or drops, we considered these as different situations/models. Pauses combined with Pitch rises or drops were viewed as mixed cues (local plus global cues), and pauses combined with Pitch breaks (i.e., when the pause between contrasting pitch values was larger than 200 ms) were viewed as local cues. Thus, in total, we had seven models.

Virtual segmentation points (cues) were marked for delexicalized and natural speech versions separately, leading to version-specific segmentation models. There was not a complete overlap in the number of segmentation points across the two versions, which was due to the audio-to-MIDI conversion process (e.g., pause lengths became slightly different in some cases, making the number of pause points differ). However, such differences were irrelevant to our main research question, which concerned the influence of instruction on the delexicalization effect rather than the delexicalization effect itself.

### 2.5. Preprocessing and Statistical Analysis

We were interested in the interaction between delexicalization (delexicalized vs. natural speech, within-subjects) and instruction (speech vs. music, between-subjects) on segmentation maps. Such interactions would indicate music-specific expectations, non-overlapping with prosody-specific ones. We analyzed the effects of delexicalization and instruction; first on segment length, and then on the adherence to a number of segmentation models we created (model matching).

To compute participants’ segment length, we calculated the interval between participants’ key presses. Participants’ metrics per stimulus (mean and standard deviation of segment length – the latter indexing segment length variability) were obtained.

**Figure 1:**
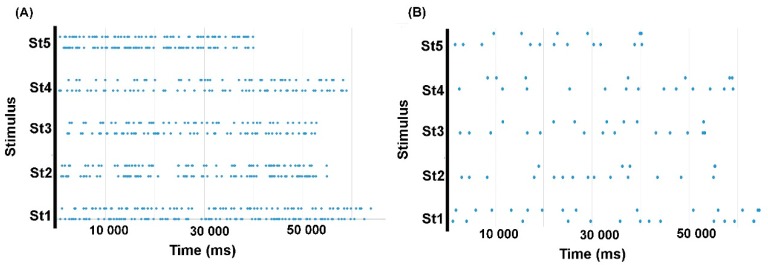
(A) Stimulus-specific global arrays, combining all segmentation models for each utterance (St1-5: Stimulus 1–5; lower line: delexicalized; upper line: natural). Dots represent virtual segmentation points; (B) Example of a segmentation maps (Participant 01) containing actual segmentation marks for each of the five stimuli in natural speech versions (above) and delexicalized ones (below, speech prosody instruction in this case).

To analyze the matching of participants’ segmentations with the segmentation models, participant-specific reaction times (see procedure; *M* + *SD* = 287 ± 50 ms) were first subtracted from the raw time of key presses in order to obtain corrected segmentation points for each stimulus (Figure 1B). Then, also for each stimulus, we merged the time stamps of the virtual segmentation points from all seven models into one global array of time values (Figure 1A).

With reference to each stimulus-specific global array of time values (lengths ranging from 45 to 96 virtual points depending on the stimulus, see Figure 1A), we derived separate logical arrays (1, true or present vs. 0, false or absent) for each model (1 marking the points of the model in question and 0 the points of other models), and one logical array per participant (1 marking participants’ segmentation points and 0 absence of segmentation). When defining participants’ logical arrays, the closest value of the global array of time values was always chosen. Maximum inter-point distances in global arrays of time values were 2690 and 3497 ms for stimulus 1 (delexicalized and natural), 4099 and 5116 ms for stimulus 2, 2397 and 3091 ms for stimulus 3, 2520 and 4886 ms for stimulus 4, 2075 and 1972 for stimulus 5. Therefore, this was the maximum error that could occur when fitting participants’ marks to the available models. Finally, we computed the similarity between the logical array describing each participant’s behavior and each of the seven logical arrays describing each model, using the Russell and Rao binary similarity coefficient [36]. The Russell and Rao coefficient evaluates the overlap of two data series concerning a binary attribute (present or absent). In our case, we measured how the distribution of participants’ marks in time overlapped with the distribution of model-specific segmentation points; both filled with present vs. absent points in reference to the global array of time values. We referred to these coefficients as model matching scores, since they described participants’ level of adherence to a given segmentation model.

For statistical analyses, we used mixed ANOVAs. We first analyzed the effects of delexicalization and instruction on the mean and standard deviation of segment length. We then considered the effects of delexicalization, instruction and model (within-subjects, seven levels/models: Pause, Pitch break, Pause plus pitch break, Pitch rise, Pitch drop, Pause plus pitch rise, Pause plus pitch drop) on model matching scores. In the presence of third-order interactions (delexicalization x instruction x model), delexicalization x instruction interactions were considered per model. Along the model matching analysis with seven models, we inspected whether the results fitted with the high-order classification of cues into local, global and mixed, to see whether it made sense to quantify the differences related to this triad. Mixed ANOVAs were also used to analyze questionnaire responses related to participants’ confidence in their segmentation responses.

Even though participants heard delexicalized versions prior to natural speech, natural speech was the common reference against which the segmentation maps of the two delexicalized conditions (speech vs. music instruction) were evaluated. Therefore, we refer to the concept of delexicalization throughout the results section as a logical, rather than chronological process.

## 3. Results

### 3.1. Segment Length

**Figure 2:**
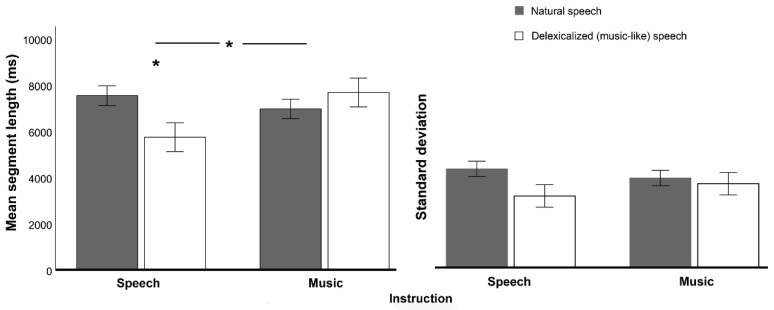
Delexicalization and instruction effects on the mean (left) and standard deviation (right) of segment length. Participants under the speech instruction decreased segment length in delexicalized versions, while those under the music instruction did not show any change. The standard deviation (variability) decreased in delexicalized versions for both instruction levels. Vertical bars represent the standard error of the mean.

The overall mean segment length was around 7000 ms (Figure 2), corresponding to an average of 8.6 segments per speech/music 60-sec sample (10.4/7.8 segments for delexicalized speech under speech/music instructions; 7.5/ 8.6 segments for natural speech). Mean segment length showed no main effects of delexicalization (*p* >0.17, η^2^p = 0.027) or instruction (*p* >0.29, η^2^p = 0.016), but there was an interaction between the two (*F*(1,68) = 10.12, *p* = 0.002, η^2^p = 0.13, Figure 2): delexicalization led to decreased segment length under the speech instruction (*t*(34) = −4.66, *p* <0.001, *d* = −0.66), while it caused no significant changes under the music instruction (*p* >0.30, *d* = 0.21, Figure 2).

Delexicalization decreased the standard deviation (variability) of segment length (main effect of delexicalization: *F*(1,68) = 4.60, *p* = 0.036, η^2^p = 0.063, Figure 2), regardless of the instruction (non- significant delexicalization x instruction interaction: *p* >0.16, η^2^p = 0.029).

### 3.2. Model Matching Scores

**Figure 3:**
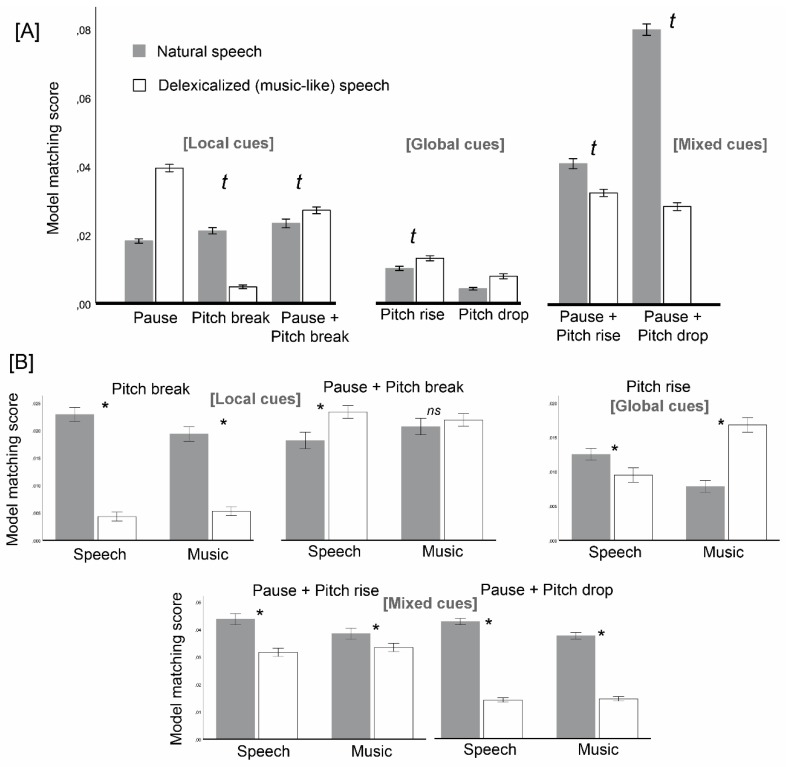
Effects of delexicalization (natural vs. delexicalized speech), model (7 models) and instruction (speech prosody vs. music) on model matching. A: Delexicalization effects per model, with five out of seven models (Pitch break, Pause + Pitch break, Pitch rise, Pause + Pitch rise, Pause + Pitch drop) showing delexicalization x instruction interactions (marked with *t*). B: Delexicalization x instruction interactions per model. Vertical bars represent the standard error of the mean.

We found a significant interaction between delexicalization and instruction (*F*(1,68) = 19.32, *p* <0.001, η^2^p = 0.22) on model matching scores. Both instruction conditions decreased general adherence to (all) models when given delexicalized versions (speech: *F*(1,34) = 99.84, *p* <0.001, η^2^p = 0.75; music: *F*(1,34) = 10.48, *p* = 0.003, η^2^p = 0.24), but the decrease was larger for the speech-prosody instruction. These effects came along with a significant (third-order) delexicalization x instruction x model interaction (Figure 3A, *F*(6,408) = 6.09, *p* <0.001, η^2^p = 0.08), suggesting that delexicalization x instruction interactions differed across models.

When the three-way interaction was broken down into the seven models (Figure 3A,B), the pattern of effects and interactions (delexicalization x instruction) was indeed heterogeneous, and it did not overlap with the associated cue types (local, global, mixed). Pauses alone (local cue, *p* >0.45, η^2^p = 0.008) and Pitch drops (global, *p* >0.95, η^2^p = 0.000) showed non-significant interactions between delexicalization and instruction. For these, delexicalization increased model matching in both instruction levels (main effect of delexicalization on matching with Pauses: *F*(1,68) = 378.96, *p* <0.001, η^2^p = 0.85; on matching with Pitch drop: *F*(1,68) = 19.16, *p* <0.001, η^2^p = 0.22). Significant delexicalization x instruction interactions showed up for Pitch breaks (local cue, *F*(1,68) = 5.15, *p* = 0.026, η^2^p = 0.07), Pitch rise (global, *F*(1,68) = 48.89, *p* <0.001, η^2^p = 0.42), Pause + pitch rise (mixed, *F*(1,68) = 4.56, *p* = 0.036, η^2^p = 0.06), and Pause + pitch drop (mixed, *F*(1,68) = 11.31, *p* = 0.001, η^2^p = 0.14). The interaction for Pause + pitch break was marginal (local cue, *F*(1,68) = 3.07, *p* = 0.084, η^2^p = 0.04). All these interactions indicate different expectations for music compared to the speech prosody instruction.

The type of interaction was independent from cue type: we saw similar patterns for Pitch break (local cue), Pause + pitch rise and Pause + pitch drop (both mixed): for all, delexicalization had the effect of decreasing model matching scores for both speech and music, with a stronger effect in speech (Pitch break: *t*(34) = 11.53, *p* <0.001, *d* = 2.69 speech, *t*(34) = 11.82, *p* <0.001, *d* = 2.56 music; Pause + pitch rise: *t*(34) = 5.41, *p* <0.001, *d* = 1.05 speech, *t*(34) = 2.04, *p* = 0.049, *d* = 0.53 music; Pause + pitch drop: *t*(34) = 27.58, *p* <0.001, *d* = 4.87 speech, *t*(34) = 17.81, *p* <0.001, *d* = 3.97 music). For Pause + pitch break—a local cue, just like Pitch break alone - delexicalization increased model matching for speech (*t*(34) = −3.35, *p* = 0.002, *d* = −0.71) while having no effect for music (*t*(34) = −0.72, *p* >0.47, *d* = −0.14). Finally, for Pitch rise – a global cue, just like Pitch drop, which showed no interaction - delexicalized versions decreased model matching scores under the speech instruction (*t*(34) = 2.47, *p* = 0.019, *d* = 0.61), while increasing it under the music one (*t*(34) = −7.46, *p* <0.001, *d* = −1.46; Figure 3B).

### 3.3. Confidence in Segmentation

Participants’ level of confidence in their segmentation responses was higher for natural speech (*M ± SD*: 3.83 ± 0.55, 5-point scale) compared to delexicalized versions (2.89 ± 0.66; *F*(1,64) = 89.61, *p* <0.001, η^2^p = 0.58). Both speech prosody and music-instruction participants showed similar gains in confidence when going from delexicalized to natural speech (*p* = 0.57, η^2^p = 0.01).

## 4. Discussion

Our goal was to determine whether the segmentation of music into phrases is driven by music-specific expectations, which would indicate that segmentation processes in music do not overlap with those that occur in speech prosody. To that end, we tested whether participants’ segmentations of a single set of ambiguous stimuli without lexical content differed according only to the identity assigned to these stimuli (speech prosody vs. music), under a manipulation of the variable instruction. Since the effect of instruction was obtained from two different groups that could be imperfectly matched, we created a baseline-related measure of this effect: we focused on how instruction influenced the within-subjects difference between natural speech (a baseline or common reference) and ambiguous, delexicalized stimuli subject to manipulations of instruction. This within-subjects difference was named delexicalization effect. In our analysis, cross-group differences in the segmentation of the natural-speech baseline were indeed apparent (see Figure 2 and Figure 3B), suggesting that participants’ segmentation strategies differed a priori across groups and thus our baseline-related measure of instruction effects was prudent.

Supporting the hypothesis of music-specific expectations, the delexicalization effect changed according to instruction in several aspects. Instruction influenced delexicalization effects on segment length: mean segment length decreased with delexicalization for the speech instruction, but it did not change for the music one. In addition, instruction changed delexicalization effects on the matching of segmentation maps with five out of seven theoretical segmentation models: for instance, the matching with Pitch rise models decreased with delexicalization for speech instruction, but it increased for music instruction (Figure 3B).

Our primary goal was to determine whether expectations in music segmentation *differed* from those in the speech-prosody domain, and thus we were interested in *any* interactions between delexicalization and instruction. Based on previous literature, we admitted the possibility that music instructions would increase reliance on global cues but, beyond that, our approach was exploratory regarding the contents of music-specific expectations. Note that, in the context of our delexicalization-effect-based approach, expectations must be framed in relative terms, i.e., how participants in each level of instruction diverged from natural speech when confronted with delexicalized versions.

The hypothesis that music segmentation would favor global cues (Pitch drop and Pitch rise) did not get support. It was true that participants under the music instruction favored Pitch rise (global cue), while those under the speech instruction devalued Pitch rise. However, the same did not go for Pitch drop, which is also a global cue. Critically, music participants favored local cues (Pitch break) and mixed cues (Pause plus pitch drop, Pause plus pitch rise) more than speech participants. Therefore, the dichotomy global–local seems irrelevant to distinguish between music and speech prosody segmentation.

Having excluded the global-cue hypothesis on music segmentation, what were we left with? First, the music instruction seems to have preserved the mechanisms of natural speech segmentation more than the speech prosody instruction: unlike speech prosody participants, music participants did not decrease segment length with delexicalization. They also preserved Pitch breaks, Pauses plus pitch drops and Pauses plus pitch rises more than speech prosody participants. The possibility that natural speech expectations may be more similar to music than prosody-specific expectations themselves is an intriguing finding that deserves further discussion. Explanations for this finding may generally relate to the disturbing potential of delexicalized speech prosody stimuli. One possibility may be that speech prosody requires phonetic content to be fully decoded, while the same does not apply to music. This might relate to the phonetic advantage effect, according to which it is easier to imitate prosody-related pitch when prosody is accompanied by phonetic content [37]. Although the authors also found a phonetic advantage for music, contradictory evidence is available [38]. In the context of our study, it is possible that dissociating speech prosody from its original phonetic content (delexicalized speech prosody versions) may have disturbed prosodic segmentation to such an extent that delexicalized music versions remained closer to natural speech. Specifically, it is possible that such disturbance was caused and/or amplified by the violation of expectations that takes place when a linguistic stimulus presents itself deprived of phonetic content. An alternative possibility may relate to the characteristics of our stimuli, namely the music-like characteristic of our delexicalized versions. We tested delexicalized stimuli using discontinuous (musical) pitch, resulting from the audio-to-MIDI transformation. Although this was necessary to maximize the credibility of the music instruction while keeping the stimuli unchanged across instruction levels, this may have created a sense of strangeness in participants from the speech group. As a result, it is possible that speech participants did not activate the bottom-up approach that we expected for speech prosody, nor the music-like set of expectations. So, although our results make it clear that speech prosody was approached differently from music, it is possible that speech prosody may have been perceived as an undetermined, unfamiliar type of auditory stream, eliciting hybrid, atypical and/or unstable expectations. In order to rule out any limitations brought by the music-like character of delexicalized stimuli - it may be helpful to add control conditions in future studies, wherein participants in each instruction condition are also presented with continuous pitch versions as delexicalized stimuli. Although these two possibilities, delexicalized speech prosody is generally disturbing, or/and particularly disturbing with discontinuous pitch, make sense, we should bear in mind that participants from the two instruction conditions did not differ in their confidence level regarding segmentation responses. From this viewpoint, one might think participants in the speech prosody instruction condition were, at least consciously, not more disturbed than those in the music condition. Still, it is possible that confidence may go along with changes in processing modes, and this may have occurred with speech prosody participants as they went from natural speech to delexicalized versions.

A second manifestation of music-specific segmentation was the increased adherence to pitch rise cues, with the opposite trend observed for speech prosody segmentation. Pitch drop is a universal, default feature of human speech [39], possibly because it is a natural outcome of decreased air flow as one vocalizes continuously without a specific pitch plan. Differently, pitch rises require planning and resources to be executed. This type of vocal attitude is characteristic of music, and it is not surprising that we saw increased expectations for pitch rise under the music instruction. Finally, music participants were unreactive to Pauses plus pitch breaks, unlike speech prosody participants, who relied more heavily on these after delexicalization. One possibility may be that the coexistence of pauses and pitch breaks tend to be interpreted more as the ending of a musical section rather than as the ending of a phrase, driving music participants to ignore this type of boundary cues. Further studies could test this possibility, by eliciting both types of segmentation; sections vs. phrases.

Concerning general aspects of our study that may deserve investigation in future studies, one relates to the order of presentation of natural vs. delexicalized versions, which may raise concerns over priming effects. In our study, participants were first exposed to delexicalized versions, and then to the natural speech counterparts. We did this because we were concerned that music-instruction participants might raise hypotheses on the origin of the delexicalized stimuli in case we had done the reverse and started with natural speech, and we wanted to avoid the risk of having to eliminate participants due to such awareness. Our choice for the present study may have introduced priming effects, but the reverse option would have done the same. Critically, potential priming effects of delexicalized over natural speech versions were common to both instruction levels, and this was all that we had to control for in face of our research question (does instruction influence the delexicalization effect?). Although the order of presentation was not likely responsible for the differences between instruction levels, it may have affected the type of expectations that were observed. This is the reason why it might be useful to counterbalance the order of block (natural vs. delexicalized stimuli) presentation in future studies. Another aspect concerns the variety of segmentation models we used, which is not exhaustive and may be expanded. Specifically, future studies may benefit from considering pre-boundary lengthening phenomena [27], which are known to guide segmentation in music and speech prosody, but which we did not consider here.

Our main finding was that there are music-specific expectations, or top-down influences, in music segmentation. Our results suggest that there is a “music-segmentation mode”, different from the processing mode engaged in speech prosody segmentation, which we assumed to be a bottom-up, data-driven approach. Although we found support for different modes, our findings do not inform us on whether speech prosody engages any expectations at all: it may be the case that music segmentation recruits expectations, or top-down processing, but speech prosody does not (our working assumption), but it may also be true that speech prosody also engages expectations, even though different from those engaged in music. A third scenario could be that speech prosody engages expectations while music segmentation is purely bottom-up, but this would go against evidence that listeners rely on metric, structural cues, such as 4-bar phrases, to perform segmentation (See Introduction Section). The best way to address these questions is to better specify the type of expectations in each domain and find cross-studies replicable patterns.

Our main finding arose from an experimental paradigm that approached music and speech prosody in ways that may not be considered fully representative of these phenomena. To probe music segmentation, we used a series of rhythmically organized discontinuous pitches without tonal organization (pitches did not organize according to tonal harmony [40]) and conveyed by a musical timbre (bass). While this approach captures basic elements of what is considered “music” (discontinuous pitch, rhythm, musical timbre), it misses important elements of common-practice music, namely tonal harmony (implicit in tonal melodies) and metric regularity. From this viewpoint, we should admit that we did not probe music segmentation in a broad sense but, rather, the segmentation of a particular music style, likely similar to contemporary jazz music (as we told our participants). So, what would happen if we used mainstream music, in case it would be possible with our paradigm? Our guess is that music-specific expectations would be more salient, since both tonal harmony and metric regularity, both absent in speech prosody, work as music-related segmentation cues [41]. In this sense, the limitations of the stimuli we presented as music concerning the ecological validity of our findings may not be significant. As for the ways we probed speech prosody, these may have limited the generality of our conclusions, as we already discussed.

## 5. Conclusions

In sum, our study was novel in testing for music-specific expectations in music segmentation, and we found evidence for these within the frame of our paradigm and assumptions. The existence of music-specific expectations in segmentation remained underacknowledged in the field of music-language comparative studies on segmentation [14,20,24]. Our findings contribute to challenge the analogy between speech prosody and music that has remained implicit in the field, setting the stage for a “music mode” and a “speech prosody mode” in segmentation.

## Figures and Tables

**Table 1:** Acoustic properties of the five stimuli used in the experiment.

	*M* ± *SD* Pitch in Hz Rel *SD*^a^ Hz/Rel *SD* Mel^b^ −1/2 *SD*, +1/3 in Mel		Pitch Change Rate (Pitches per Second)	Duration (sec)	Silence Proportion (%)	
	Natural	Delexicalized	Delexicalized ^c^		Natural	Delexicalized
1	185 ± 28 Hz 0.55/−0.53, +0.55 −18, +12	187 ± 26 Hz 0.51/−0.50, +0.50 −17, +11	3.47	63.4	44.7	41.7
2	112 ± 21 0.41/−0.43, +0.45 −14, +10	112±19 0.37/−0.38, +0.40 −13, +9	3.2	55.5	4.5	19.6
3	130 ± 35 0.69/−0.72, +0.73 −26, +16	128 ± 34 0.67/−0.69, +0.72 −12, +15	3.02	57.0	32.4	34.5
4	172 ± 51 1/−1, +1 −34, +21	170 ± 51 1/−1, +1 −34, +22	3.09	59.1	20.1	24.6
5	123 ± 20 0.39/−0.40, +0.42 −13, +9	123 ± 18 0.35/−0.35, +.39 −12, +9	4	40.2	23.7	30.8

^a^ Relative SD = SD/highest SD (Stimulus 4). Note that the magnitude relation across stimuli is equivalent, whether it comes in Hz or in Mel; ^b^ Mel – Measure of pitch that accounts for different sensitivity levels across the frequency range; ^c^ in natural speech, pitch change is continuous.

**Figure A1:**
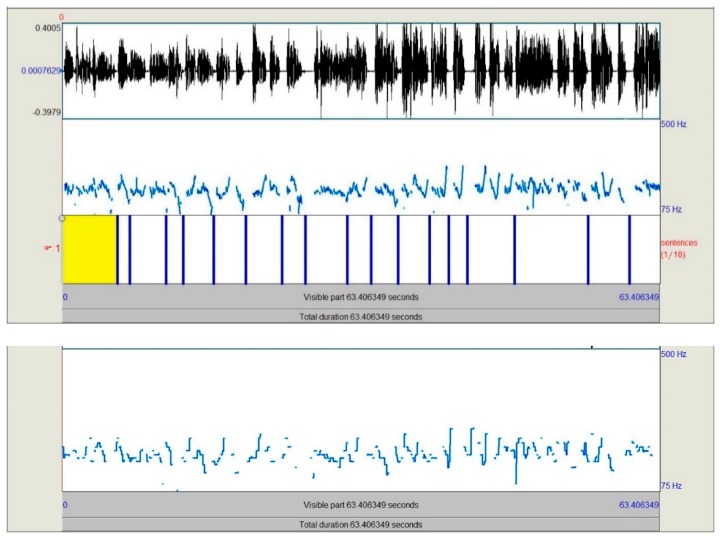
Stimulus 1—Pitch structure.

**Figure A2:**
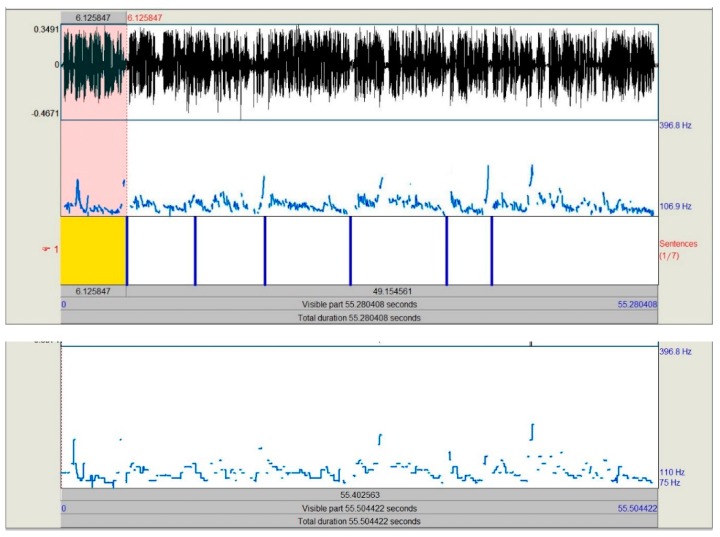
Stimulus 2—Pitch structure.

**Figure A3:**
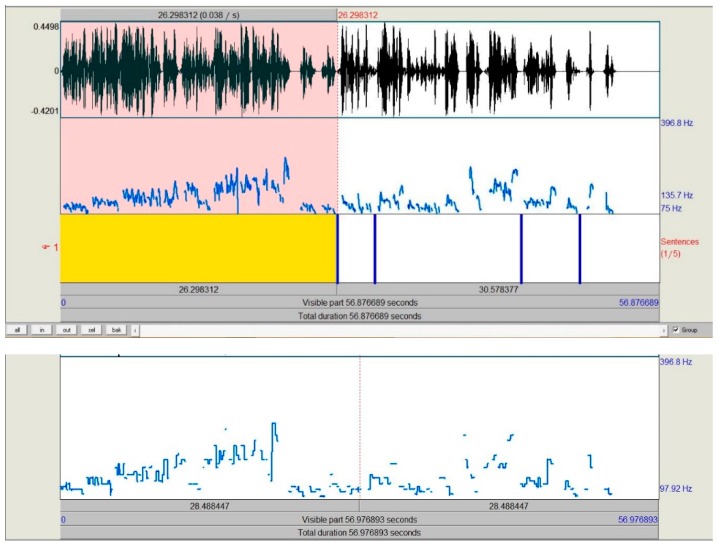
Stimulus 3—Pitch structure.

**Figure A4:**
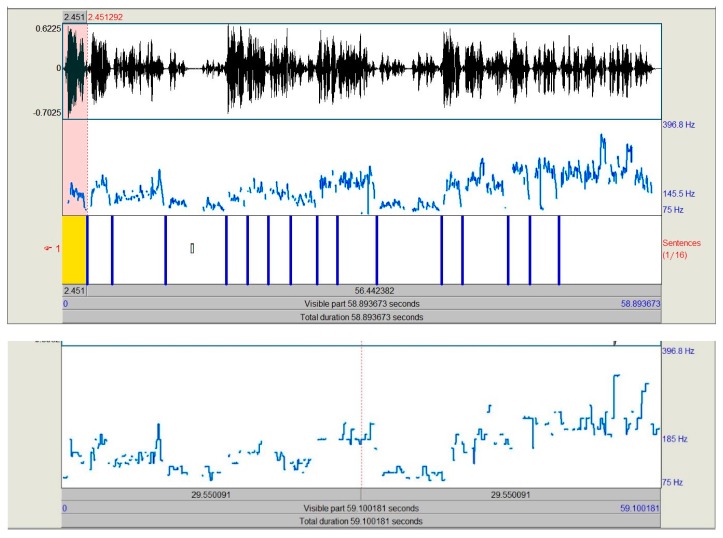
Stimulus 4—Pitch structure.

**Figure A5:**
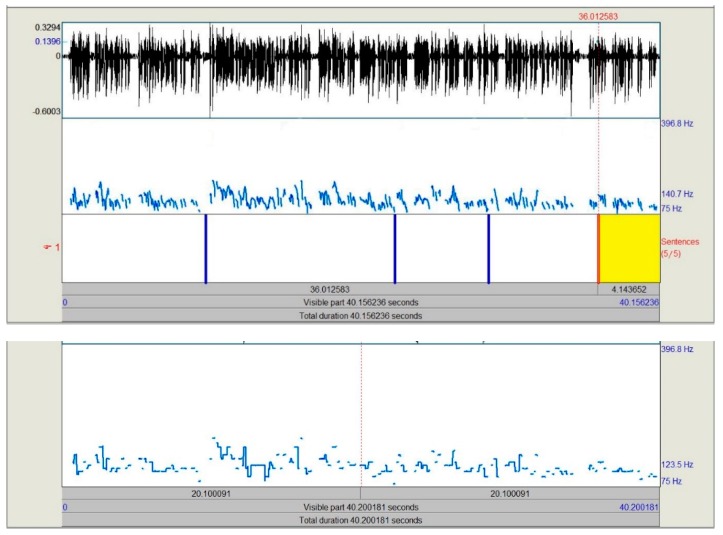
Stimulus 5—Pitch structure.

## Supplementary Materials

Stimulus materials can be downloaded from: https://drive.google.com/file/d/1XGyxhRsByrcTmymHiCNnPRcCegwYYIoo/view?usp=sharing.

## Appendix A

Pitch Structure and Linguistic Content of the Five Stimuli

In each figure, pitch structure is plotted above for natural speech stimuli, and below for delexicalized versions. Boundaries under natural speech versions indicate segmentation based on linguistic context, specifically on sentences as defined in transcription texts.

In transcriptions, // indicates clause boundaries,** bold and underlined **words indicate the main verb, and underlined words the verb (and conjunctions) in subordinate and/or coordinate clauses. Self-corrections and hums are included.

**Table A1:** Stimulus 1—Transcription.

Portuguese	English
O gravador… com os bichinhos todos, br- brinquedos, um cãozinho **anda** à volta dos brinquedos. // Os brinquedos **são** animaizinhos: // **Tem** uma boneca, um pintainho... uma foca, // e o cãozinho anda à volta deles. // Olha, **começaram a andar**, os bichinhos. // **Ficou** só o, a andar à volta à beira do gravador. // A mão **veio**, // apanhou o carro... // e amassou-o. // **Veio** agora uma rãzinha... e uma bonequinha. // A bonequinha **foi amassada**. // E foi... com o pincel, **varreu** a bonequinha para o lado, para ela desaparecer. // **Veio** o pintainho, // [e] passou-lhe a bola por cima… // Com o apanhador e com a vassoura, **apanharam **o pintainho. // Depois **veio** outro patinho, // [e] também levou com o apanhador... // Outro bonequinho **foi** com a mão... // **Apanhou** os dois.... // **Veio** uma foquinha... // [e] foi apanhada… a foquinha… // **Veio** agora outro bichinho, // foi varrido, // e a rãzinha virou-se. // **Cortaram-lhe** a cabeça… // tiraram-lhe a cabeça… // meteram os dedos, // [e] puxaram. // **Mostrou** dois dedos - um dedo a avisar… Três dedos. // [**Diz:**] “Futebol Clube do Porto, tricampeão, noventa e quatro, noventa e sete”. //	The recorder… with all the little animals, to- toys, a puppy **goes** around the toys. // The toys **are** little animals: // **There’s **a doll, a chick… a seal, // and the puppy goes around them. // Look, they** started to walk**, the animals. // Only the car** stayed**, going around next to the recorder. // The hand **came**, // caught the car… // and mashed it. // Just now **came** a little frog… and a little doll. // The little doll **was mashed**. // And went... with the paintbrush, **brushed** the little doll to the side to make her disappear. // The chick **came**, // [and] the ball passed over it… // With the dustpan and the broom, they **got** the chick. // Then **came **another little duck, // [and] the dustpan also hit it... // Another little doll**was** with the hand… // It **caught**both… // Then a little seal **came**… // [and] it was caught… the little seal… // Now another little animal **came**, // it was brushed away, // and the little frog turned, // They **cut off** its head… // took off its head… // put their fingers in, // [and] pulled. // It showed two fingers – one finger warning… Three fingers. // [**It says:**] Porto Football Club, tri-champion, ninety four, ninety seven. //

**Table A2:** Stimulus 2 - Transcription.

Portuguese	English
**Era** um grupo muito organizado, hã, desde a base até, hã... ao topo. // hã, Um grupo que, numa primeira fase… // como eu referi, //**é** um grupo de estrangeiros. // Alguns deles, hã… no passado, já **foram** funcionários de empresas // que prestam serviços à Portugal Telecom. // hã- E, como tal,**tinham** acesso, muitas vezes privilegiado, a localização de- de- de linhas inativas da- da Portugal Telecom. // hã- Eles, identificando, digamos assim, estas localizações… // **procediam** então ao furto, hã, de determinados troços, hã, das linhas de cobre. // Para tal,** recorriam**, hã, à utilização de viaturas tipo furgon… // [As] viaturas estas [**estavam**] preparadas com um alçapão, hã, no seu fundo… que colocavam em cima das caixas, procedendo então à entrada nas respetivas caixas e ao corte de pequenos troços destas linhas // que encaminhavam para- que encaminhavam para um interior da- da viatura. //	It **was** a very organized group, uhh, from the base to, uhh... the top. // uhh A group that, in a first phase… // as I mentioned, // **is** a group of foreigners. // Some of them, uhh… in the past,** had been** employees of companies // that provide services to Portugal Telecom. // uhh- And, as such, they **had** access, often privileged, to the location of- of- of inactive lines of- of Portugal Telecom. // uhh- They, by identifying, as it were, these locations… // they **would proceed** to the theft, uhh, of specific sections, uhh, of the copper lines. // For this, they **resorted,** uhh, to the use of vehicles like Furgon vans… // Vehicles [**were**] prepared with a trapdoor, uhh, in its bottom… that they would place on top of the boxes, // proceeding then to enter in the respective boxes and to cut small sections of these lines, // that they redirected to- they redirected to the inside of- of the vehicle. //

**Table A3:** Stimulus 3—Transcription.

Portuguese	English
Se vivendo entre o povo és virtuoso e nobre, //se vivendo entre os reis conservas a humildade, // se inimigo ou amigo, o poderoso e o pobre, são iguais para ti, à luz da eternidade, // se quem conta contigo // encontra mais que a conta, // se podes empregar os sessenta segundos do minuto //que passa// em obra de tal monta que o minuto se espraie em séculos fecundos,// então, ó ser sublime, o mundo inteiro **é** teu. // Já **dominaste** os reis, os espaços! // Mas, ainda para além, um novo sol**rompeu**, abrindo o infinito aos rumos dos teus passos, pairando numa esfera... acima deste plano... // sem recear jamais // que os erros te retomem… // Quando já nada houver em ti // que seja humano… // **alegra-te**, meu filho. // Então... **serás** um homem! //	If while living amongst the people // you are virtuous and noble, // if while living amongst kings // you keep your humility, // if foe or friend, powerful or poor, are equal to you in the light of eternity, //If the ones who count on you // find more than the bill, //if you can fill the sixty seconds of the minute // that passes in work of such amount, // in a way that the minute spreads in fertile centuries, // then, oh sublime being, the whole world** is** yours. // You’**ve conquered **kings, spaces! // Yet, still beyond, a new sun **has risen,** opening infinity towards your steps, hovering in a sphere… above this plane... //never fearing // that mistakes return to you… // When there is nothing left in you that is human… //**rejoice**, my son. // Then…you**will be** a man. //

**Table A4:** Stimulus 4—Transcription.

Portuguese	English
À roda da nau**voou**três vezes. // **Voou** três vezes a chiar // e disse: // “Quem é que **ousou entrar** nas minhas cavernas //que não desvendo, meus tetos negros do fim do mundo?”// E o homem do leme**disse**, tremendo: “El-Rei D. João Segundo”. // “De quem **são** as velas // onde me roço? // De quem [**são**] as quilhas // que vejo // e ouço?”// **Disse**o mostrengo // e rodou três vezes. // Três vezes**rodou**, imundo e grosso. // “Quem **vem pode**r // o que só eu posso? // [Eu] que **moro** // onde nunca ninguém me visse // e escorro os medos do mar sem fundo?” // E o homem do leme**tremeu** // e disse: // “El-Rei D. João Segundo”. // Três vezes do leme as mãos**ergueu. **// Três vezes ao leme as**reprendeu**, // e disse no fim de tremer três vezes: // “Aqui ao leme **sou** mais do que eu! // **Sou** um povo //que quer o mar que é teu! // E mais que o mostrengo //que me a alma teme // e roda nas trevas do fim, // **manda** a vontade //que me ata ao leme// de El Rei D. João Segundo!” //	Around the ship’s wheel it**flew** three times, // It **flew** three times creaking // and said: // “Who **has dared** to enter my caverns // that I don’t unravel, my dark ceilings of the end of the world?” // And the helmsman **said**, trembling: “The King D. João the Second”. // “Whose** are** the sails // I’m rubbing? // Whose **are** the keels //that I see // and hear?”// The moster** said** // and turned three times. // Three times he** turned**, filthy and thick. // “Who **comes** to do // what only I can do? // I** live** // where no one has ever seen me // and I drain the fears of the bottomless sea?” // And the helmsman **trembled** // and said: // “The King D. João the Second”. // Three times from the helm his hands **were raised. **// Three times to the helm they w**ere reattached,** // and said he after trembling three times: // “Here at the helm **I am** more than myself! // I **am** a people that wants the sea that is yours! // And more than the monster // that my soul fears, // and turns in the darkness at the end, // the will that commands and ties me to the helm **is** the one of King D. João the Second!” //

**Table A5:** Stimulus 5—Transcription.

Portuguese	English
A pedido da direção do canal, e depois do arranque do programa Curto Circuito,**é-nos solicitada **a divulgação de um comunicado, // que passo a citar: // “Após ter analisado em direto o conteúdo do programa denominado Curto Circuito, // a direção da SIC Radical **decidiu****romper** unilateralmente o contrato // que a ligava à produtora Sigma 3. // Com esta medida, o programa denominado Curto Circuito **é retirado** imediatamente de grelha.” // **Assina** o diretor do canal, Francisco Penim, // que está comigo em estúdio para clarificar… esta posição. // Francisco, boa tarde. A pergunta não **pode** ser outra: Porquê… esta decisão? //	By request of the board of the channel, and after the beginning of the show Curto Circuito, we **are asked** to divulge a bulletin // that I hereby quote: // “After having analyzed the content of the show called Curto Circuito, // the board of SIC Radical has **decided to** unilaterally **break **the contract that connected it to the producer Sigma 3. // With this action, the show called Curto Circuito **is** immediately** withdrawn** from the grid.” // It is signed by the channel’s director, Francisco Penim, // **who is **with me in the studio to clarify… this position. // Francisco, good afternoon. The question **cannot be** other: Why… this decision? //
